# Bilateral Hegu Acupoints Have the Same Effect on the Heart Rate Variability of the Healthy Subjects

**DOI:** 10.1155/2014/106940

**Published:** 2014-06-26

**Authors:** Wang Guangjun, Tian Yuying, Jia Shuyong, Zhou Wenting, Zhang Weibo

**Affiliations:** Institute of Acupuncture and Moxibustion, China Academy of Chinese Medical Sciences, Beijing 100700, China

## Abstract

*Background*. The specificity of acupuncture points (acupoints) is one of the key concepts in traditional acupuncture theory, but the question of whether there is adequate scientific evidence to prove or disprove specificity has been vigorously debated in recent years. Acupoint laterality is an important aspect of acupoint specificity. Data is particularly scarce regarding the laterality of the same channel, namesake acupoint located on opposite sides of the body. Our previous study results suggest that Neiguan acupoint (PC6) has the laterality. The aim of this study was to investigate whether Hegu (LI4) also has laterality from the perspective of heart rate variability. *Methods*. A total of twenty-eight healthy female volunteers were recruited for this study and were randomly separated into the group I (*n* = 14) and the group II (*n* = 14) according to the register order. In the group I, left LI4 was stimulated in the first epoch and the right LI4 was stimulated in the second epoch. In the group II, right LI4 was stimulated in the first epoch and left LI4 was stimulated in the second epoch. Electrocardiogram was recorded and heart rate variability was analyzed. *Results*. The results show that there were no significant differences of heart rate variablity between the group I and the group II in the time domain and in the frequency domain. *Conclusions*. Bilateral Hegu acupoints have the same effect on the heart rate variability of the healthy subjects.

## 1. Background

In the traditional Chinese medicine (TCM) theory, acupoint specificity is an essential principle [1, 2] which means that the therapeutic efficacy mainly resulted from the correct acupoint choice. However, it is still not clear whether right or left acupoint should be selected for the purpose of treating a particular disease. From the perspective of acupuncture analgesia, stimulation of specific acupuncture points will promote the release of analgesic substances in the central nervous system such as opioid peptides [3, 4], and the targets of these substances locate in the central nervous system and result in the systemic analgesic effect, which means that the bilateral acupoints might have the same effect. On the other hand, there are such records as “if someone has disease related with the left side, the treatment point is the right side, and vice versa,” “*巨刺*” and “*缪刺*” in the Yellow Emperor Neijing, which emphasized that specific lateral side acupoint stimulation might lead to the therapeutic advantages under the specific conditions. Particularly, this idea was developed into the concept of contralateral acupuncture in recent years [5, 6] and we summarized the acupoint laterality [7]. From our previous study, we found that ipsilateral stimulation of Hegu (LI4) corresponded to the increased blood perfusion in the contralateral Hegu (LI4) [8]. Moreover, the increased degree of blood perfusion was asymmetrical, which suggested that the LI4 has the laterality in the perspective of blood distribution [9]. Further study indicated that acupuncture different side Neiguan (PC6) had the different effect on the heart rate variability, which means that PC6 also has the laterality [10]. Recently, the Quchi (LI11) effects on the HRV in patients with hypertension were investigated and the results partly supported our result [11]. However, there is still scarcity of the evidence that every acupoint has the laterality from the perspective of HRV modulation. The aim of this study is to explore whether different side LI4 stimulation can result in the different effect on the heart rate variability.

## 2. Materials and Methods

### 2.1. Ethics Statement

This study was reviewed and approved by the Institutional Review Board at the Institute of Acupuncture and Moxibustion, China Academy of Chinese Medical Sciences. Each participant read and signed an informed consent form.

### 2.2. Subjects and Design

Twenty-eight (28) healthy female volunteers were recruited in this study. All subjects were students from the China Academy of Chinese Medical Sciences and Beijing University of TCM. None of the subjects had a history of prior disease nor had they taken any medication in the six months prior to the study. Each subject was provided with informed consent and had an adequate understanding of the procedure and purpose of this study. Basic characteristics of the participants are shown in Table 1. The subjects were randomly separated into the group I (*n* = 14) and the group II (*n* = 14) according to the register order. In the group I, left LI4 was stimulated in the first epoch and the right LI4 was stimulated in the second epoch. In the group II, right LI4 was stimulated in the first epoch and left LI4 was stimulated in the second epoch.

### 2.3. Electrocardiogram Measurement Protocol

**Figure 1:**
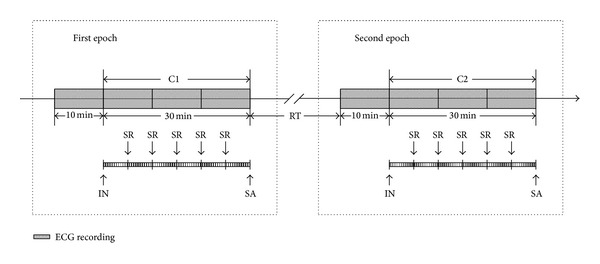
Procedure of acupuncture and measurement. ECG recording time point; IN: insert needle; SA: stop acupuncture; SR: slowly rotate the needle every 5 min. The needle was slowly rotated every 5 min for a total of 30 min during the C1 and C2 acupuncture sessions; RT: rest time between two epochs.

Before the laboratory procedure began, subjects were placed in a temperature-controlled room (24–26°C) to rest for 10 minutes. The standard lead II ECG was recorded with NeurOne system (NeurOne, MEGA electronics Ltd., Finland) [10]. The data were digitized with a sampling rate of 1000 Hz. In the first epoch, the successive ECG were recorded and symbolized as C1. In the second epoch, the successive ECG were recorded and symbolized as C2. In each epoch, a 30 min ECG recording was obtained (shown in Figure 1). The interval of two experimental epochs is at least 7 days.

### 2.4. Acupuncture Protocol

For every participant, either the right or left LI4 was stimulated during the first epoch of the study and the opposite side LI4 was stimulated during the second epoch. The interval time between the two epochs was at least 8 days. For the acupuncture procedure, a small acupuncture needle, 0.25 × 25 mm (100112, Zhen Huan), was gently inserted into a depth of 15 mm in LI4. The needle was slowly rotated every 5 min for a total of 30 min during the acupuncture session in order to maintain the soreness and numbness sensation of De-Qi [8, 12]. The acupuncture process is illustrated in the figure.

### 2.5. Data Analysis

The raw data recorded by NeurOne system was exported with ASC format and then imported into Kubio HRV software and analyzed [13]. The analysis parameter was default. In the time domain, the mean RR interval (RR), the standard deviation of RR intervals (STDRR), the root mean square of successive differences (RMSSD), the number of successive intervals differing more than 50 ms (NN50), and the corresponding relative amount of NN50 (pNN50) were analyzed. In the frequency domain, the power spectrum density was analyzed with AR spectrum method in normalized units. The low frequency (LF) and high frequency (HF) were defined as 0.04–0.15 Hz and 0.15–0.4 Hz, respectively. Data are expressed as mean ± SD. For each parameter, the difference of C1-C2 was calculated and then *t*-test was performed between group I and group II. The level of significance was defined as *P* < 0.05. Statistical analyses were performed using SPSS (SPSS Inc., Chicago, IL, USA).

## 3. Results

Table 2 presents the results of time domain analysis and frequency domain analysis. In the time domain, mean RR, STD RR, RMSSD, NN50, pNN50, SDANN, and SDNN index were analysed and there was no significant difference between the group I and the group II. In the frequency domain, the power of VLF, LF, and HF, the power percentage of VLF, LF, and HF, the normalized LF and HF, the total power, and LF/HF ratio were analyzed and there were no significant differences between the group I and the group II.

## 4. Discussion

Heart rate variability (HRV) analysis by Fourier transform is a noninvasive method to investigate autonomic balance [14]. The variance in the high frequency (HF) range (0.15–0.40) Hz is thought to primarily reflect vagal activity while the variance in the low frequency (LF) range (0.04–0.15 Hz) is influenced both by the vagus and the sympathetic nervous system [15]. When a needle is inserted into a point on the body various neural and neuroactive components are activated [16, 17]. Mechanism explore has been shown that acupuncture have the clear central nervous system and autonomic nervous system effects both in humans [18, 19] and in animals [20]. Because the electroacupuncture can mimic the direct vagus stimulation effect [21], the analysis of HRV provides quantitative information regarding autonomic control mechanisms in the body [22]; thus, HRV has recently been adopted as an index used to evaluate the effects of acupuncture [23].

Previous study indicated that the cardiac modulatory balance differs between genders and is characterized by a greater influence of the autonomic vagal component in women and by the sympathetic component in men [24]. Another study investigated the influence of age and gender on the short-term HRV indices and revealed significant modifications of the indices especially by age but partly also by gender especially in the younger groups [25]. To exclude gender bias, we only recruited the healthy adult females in our study [10].

Previous studies demonstrated that acupuncture manipulation significantly decreased the LF spectral component of HRV and significantly reduced LF/HF, which is an index of sympathetic activity [26]. Related results suggested that appropriate stimulation at the PC6 can modulate the HRV in healthy subjects [27]. In our previous study, acupuncture different side PC6 results in lateral effect, and this effect on heart rate variability mainly occurred in the acupuncture period. After acupuncture was discontinued, this effect disappeared [10].

In this study, the laterality of LI4 was explored. From the previous study, manual stimulation of Hegu (LI4) resulted in specific changes in alpha EEG frequency and in HRV parameters [28]. We originally expected that stimulate different side LI4 also can result in the different effect on HRV, but the results are apparently at odds with our prospective. One possible explanation is according to traditional acupuncture theory: PC6 is a relative specific acupoint for cardiovascular disease and is one of the most commonly used acupoints in classical texts [29–31]; however, LI4 belongs to large intestine meridian and is not sensitive to modulate the cardiovascular function.

## 5. Conclusions

Bilateral Hegu acupoints have the same effect on the heart rate variability of the healthy subjects.

## Figures and Tables

**Table 1:** Basic characteristics of the study participants.

Characters	Group I (*n* = 14)	Group II (*n* = 14)
Age (years)	26.14 ± 1.19	25.93 ± 1.39
High (cm)	164.93 ± 3.92	162.21 ± 4.35
Body weight (kg)	54.64 ± 6.96	52.64 ± 4.92
Body mass index (BMI)	18.69 ± 5.61	20.01 ± 1.7

**Table 2:** Results of heart rate variability.

	Group I (*n* = 14)		Group II (*n* = 14)		*t*	*P*
	C1	C2	C1	C2		
Mean RR (ms)	876.72 ± 90.13	843.27 ± 54.87	913.64 ± 142.68	865.18 ± 106.56	−0.5474	0.5888
STD RR (ms)	55.9 ± 16.66	54.85 ± 16.24	55.19 ± 13.26	53.62 ± 20.08	−0.1349	0.8938
RMSSD (ms)	45.93 ± 21.62	41.4 ± 17.62	45.48 ± 18.95	41.89 ± 26.63	0.1772	0.8607
NN50 (count)	464.86 ± 362.08	446 ± 371.65	476.64 ± 316.35	395.43 ± 409.93	−0.7654	0.4509
pNN50 (%)	23.48 ± 19.79	21.47 ± 18.56	25.21 ± 17.83	19.92 ± 21.39	−0.7206	0.4776
VLF (ms^2^)	1602.75 ± 925.69	1567.78 ± 1097.23	1379.81 ± 585.48	1401.52 ± 578.8	0.2157	0.8309
LF (ms^2^)	601.15 ± 389.84	586.25 ± 374.28	568.15 ± 555.34	549.14 ± 528.43	−0.0463	0.9634
HF (ms^2^)	966.4 ± 862.48	834.81 ± 713.49	1046.1 ± 823.59	1059.35 ± 1425.28	0.5885	0.5613
VLF (%)	54.53 ± 16.2	54 ± 15.25	50.17 ± 19.04	56.03 ± 16.78	1.5706	0.1284
LF (%)	18.04 ± 6.66	19.11 ± 6.8	17.8 ± 11.91	17.21 ± 5.69	−0.6633	0.513
HF (%)	27.39 ± 13.85	26.86 ± 13.96	32 ± 15.89	26.72 ± 14.51	−1.267	0.2164
LF (n.u.)	41.44 ± 12.61	44.12 ± 16.84	35.73 ± 18.11	41.17 ± 12.98	0.6232	0.5386
HF (n.u.)	58.47 ± 12.62	55.81 ± 16.84	64.2 ± 18.11	58.76 ± 12.99	−0.6254	0.5372
Total power (ms^2^)	3171.55 ± 1873.31	2989.72 ± 1860.04	2994.95 ± 1327.92	3011.03 ± 2345	0.4523	0.6548
LF/HF ratio	0.78 ± 0.37	1.01 ± 0.83	0.76 ± 0.81	0.8 ± 0.53	−0.8358	0.4109

RR: RR intervals; STDRR: standard deviation of RR intervals; RMSSD: root mean square of successive differences between RR intervals; NN50: the number of successive intervals differing more than 50 ms; pNN50: the corresponding relative amount of NN50 (pNN50); LF: low frequency; HF: high frequency; VLF: very low frequency; data are expressed as mean ± SD. For each parameter, the difference of C1-C2 was calculated and then *t*-test was performed between the group I and the group II.
